# Hospital Gazettes for July

**Published:** 1919-08-09

**Authors:** 


### HOSPITAL GAZETTES FOR JULY.

### GUY'S HOSPITAL.

THE Editor of *Guy's Hospital* expresses the opinion that one inevitable fact dependent on the coming of Peace—an important one so far as the material weal of medical graduates is concerned—is the fact that newly qualified medical men will no longer automatically step into house appointments. There will be considerable competition for these posts in future, and the majority must inevitably fail in being appointed. An agency has been started at Guy's Hospital under control of Mr. Winston, the Will's Librarian, which is run entirely for Guy's men, and should be very helpful to those who have no definite work to undertake.

### ST. THOMAS'S HOSPITAL.

THERE is an excellent article in the *St. Thomas's Hospital Gazette* on "Reconstruction in our Hospitals" which deals largely with the subject of medical examinations. In all branches of education it is well known that there are two types of students—*viz.*, those who are good at examinations, and those who, while not good at examinations, are all the same possessed of a good store of knowledge. It may be that the following paragraph in the above article has been written in the interests of the latter, who often need much sympathy. The writer asks if it is possible to modify the existing system of examinations. "A final examination by representatives of some recognised body is essential before society can license a man to practise medicine. Admitted that any such test is unsatisfactory, it remains unavoidable for those who are to be entrusted with certain duties and responsibilities by the State. The present system of continually recurring examinations is, however, simply pernicious. An examination is always looming ahead. The student measures every part of his studies by the entirely false standard of its examination value, and once he has passed the test relieves his overloaded brain by a species of mental emesis before he begins to assimilate the next dish set before him. The result is that a course of study, which to be of any value should be continuous, and in which one subject is supposed to lead on to another, becomes a series of disconnected lessons no sooner learnt than forgotten. If for the present series of examinations an entrance examination, including chemistry, physics, and possibly some general subjects, and a final examination, for qualification could be substituted, the student could pursue the even course of his studies untroubled by bouts of feverish cramming. A prolonged final examination, in which all the professional subjects had to be taken together, and in which some knowledge of physiology and surgical anatomy was demanded, would give a far better idea of a man's real capacity to practice than the present fluky system of single subjects."

